# Acute Low-Intensity Treadmill Running Upregulates the Expression of Intestinal Glucose Transporters via GLP-2 in Mice

**DOI:** 10.3390/nu13051735

**Published:** 2021-05-20

**Authors:** Kai Aoki, Takuji Suzuki, Fang Hui, Takuro Nakano, Koki Yanazawa, Masato Yonamine, Shinichiro Fujita, Takehito Sugasawa, Yasuko Yoshida, Naomi Omi, Yasushi Kawakami, Kazuhiro Takekoshi

**Affiliations:** 1 Japan Society for the Promotion of Science, Kojimachi Business Center Building, Kojimachi, Chiyoda-ku, Tokyo 102-0083, Japan; K-Aokitsuku@md.tsukuba.ac.jp; 2 Faculty of Medicine, University of Tsukuba, 1-1-1 Tennodai, Ibaraki 305-8577, Japan; osaraba4ma@gmail.com (M.Y.); shin.ichiro.fujita.03@gmail.com (S.F.); take0716@krf.biglobe.ne.jp (T.S.); y-kawa@md.tsukuba.ac.jp (Y.K.); 3 Department of Food Science and Nutrition, Doshisha Women’s College of Liberal Arts, Tera-machi Nishiiru, Imadegawa-dori, Kamigyo-ku, Kyoto 602-0893, Japan; tasuzuki@dwc.doshisha.ac.jp; 4 Graduate School of Comprehensive Human Sciences, University of Tsukuba, 1-1-1 Tennodai, Tsukuba, Ibaraki 305-8577, Japan; s2030470@s.tsukuba.ac.jp (F.H.); s2021408@s.tsukuba.ac.jp (T.N.); s1921312@s.tsukuba.ac.jp (K.Y.); 5 Department of Clinical Laboratory, Faculty of Health Sciences, Tsukuba International University, 6-20-1 Manabe, Tsuchiura, Ibaraki 300-0051, Japan; y-yoshida@tius.ac.jp; 6 Faculty of Health and Sport Sciences, University of Tsukuba, 1-1-1 Tennodai, Tsukuba, Ibaraki 305-8577, Japan; omi.naomi.gn@u.tsukuba.ac.jp

**Keywords:** low-intensity exercise, intestine, sodium-dependent glucose transporter, glucose transporter 2, glucagon-like peptide 2

## Abstract

The effects of exercise on nutrient digestion and absorption in the intestinal tract are not well understood. A few studies have reported that exercise training increases the expression of molecules involved in carbohydrate digestion and absorption. Exercise was also shown to increase the blood concentration of glucagon-like peptide-2 (GLP-2), which regulates carbohydrate digestion and absorption in the small intestine. Therefore, we investigated the effects of exercise on the expression of molecules involved in intestinal digestion and absorption, including GLP-2. Six-week-old male mice were divided into a sedentary (SED) and low-intensity exercise (LEx) group. LEx mice were required to run on a treadmill (12.5 m/min, 1 h), whereas SED mice rested. All mice were euthanized 1 h after exercise or rest, and plasma, jejunum, ileum, and colon samples were collected, followed by analysis via IHC, EIA, and immunoblotting. The levels of plasma GLP-2 and the jejunum expression of the GLP-2 receptor, sucrase-isomaltase (SI), and glucose transporter 2 (GLUT2) were higher in LEx mice. Thus, we showed that acute low-intensity exercise affects the expression of molecules involved in intestinal carbohydrate digestion and absorption via GLP-2. Our results suggest that exercise might be beneficial for small intestine function in individuals with intestinal frailty.

## 1. Introduction

Exercise has been shown to induce various physiological and biochemical changes within the body. Exercise therapy has been explored for the management of dementia, weak locomotion–associated sarcopenia, and diabetes [1,2,3,4,5,6]. Several studies have demonstrated the effects of exercise on various organs, and, more recently, its effects on the small intestine have also been reported [7,8]. Intestinal permeability and inflammation [9,10,11] and to a lesser extent digestive and absorptive capability are influenced by exercise. Digestion and absorption within the small intestine are required in order for the host organism to absorb nutrients from food. However, after clinical interventions involving fasting and total parenteral nutrition (TPN), the digestive and absorptive capability of the small intestine are remarkably compromised, a phenomenon that is associated with a decrease in the expression of nutrient digestion- and absorption-related genes [12,13]. To date, two studies have reported the effects of exercise on the expression of intestinal glucose transporters. Motiani et al. reported that two weeks of moderate-intensity training upregulated the expression of glucose transporter 2 (GLUT2) in the intestines of mice [14]. Kondo et al. reported that six weeks of swimming exercise induced substantial alterations in pancreatic digestive capability as well as in the absorptive capability of the small intestine in rats [15]. Therefore, exercise might be effective in improving the digestion and absorption of carbohydrates within the small intestine. Nevertheless, the mechanism underlying these adaptive changes remains unknown. In addition, it is important to specify the exercise intensity necessary for their induction. Exercise intensity can be established based on aerobic threshold (AT), ventilation threshold (VT), and lactate threshold. If low-intensity exercise can be performed by patients in order to enhance carbohydrate digestion and absorption within the small intestine, it will be of major clinical benefit.

The expression of carbohydrate transporters and saccharidases within the small intestine is known to be regulated via nutritional stimulation. The sodium-dependent glucose transporter (SGLT1) and GLUT2—typical glucose transporters—in addition to sucrase-isomaltase (SI), a typical disaccharidase, are sensitive to regulation via dietary saccharides, which enhance their expression. It was recently found that glucagon-like peptide-2 (GLP-2) also upregulates the expression of these proteins [16,17,18,19,20,21]. GLP-2 plays a pivotal role in the maintenance of intestinal function. Several studies on its effects in humans and animal models have suggested its therapeutic efficacy against intestinal dysregulation, as in the case of long-term TPN treatment, short bowel syndrome, and inflammatory bowel disease [22,23,24,25,26,27]. Drucker et al. demonstrated the efficacy of GLP-2 analogs in human patients [23], with *Teduglutide* currently approved as a pharmaceutical in the USA. Acute aerobic exercise has been reported to increase the plasma levels of GLP-1 [28], which is a hormone also derived from proglucagon and secreted by the same cell type as GLP-2, namely L cells. Therefore, it is conceivable that exercise might increase the plasma levels of GLP-2, thereby upregulating the expression of carbohydrate transporters and disaccharidases within the small intestine. Investigation into the exercise-induced increase in intestinal digestion and absorption of carbohydrates and the underlying mechanism would provide new insights for the implementation of exercise therapy in patients with decreased digestion and absorption capacity.

In this study, we hypothesized that exercise increases the expression of proteins associated with carbohydrate digestion and absorption via GLP-2 and tested this hypothesis using an acute low–intensity exercise mouse model. Indeed, low-intensity exercise had beneficial effects on the expression of molecules associated with carbohydrate digestion and absorption within the intestine.

## 2. Materials and Methods

### 2.1. Animal Treatment and Acute Exercise

The present study was conducted in accordance with the international principles and guidelines for animal care and was approved by the Animal Experimental Committee, University of Tsukuba (approval number: 20–492). Briefly, 6-week-old ICR male mice were purchased from CLEA Japan Inc. (Tokyo, Japan). Mice were bred and maintained in an air-conditioned animal house under specific-pathogen-free conditions and subjected to 12/12 h light and dark cycles. Animals had ad libitum access to standard food pellets and water. At 7 weeks of age, all mice were familiarized with treadmill running for 20–30 min per day for three days. Then, the mice were divided into a sedentary (SED; *n* = 6) or low-intensity exercise (LEx; *n* = 6) group and allowed to rest for 24 h. Mice in the LEx group were subjected to acute treadmill running for 1 h at a rate of 12.5 m/min. Mice were subjected to fasting for 2 h before exercise. After acute exercise, mice were allowed to rest for 1 h and were then euthanized. Mice in the SED group were euthanized after 2 h of fasting followed by a rest period.

### 2.2. Tissue Sampling

Mice were euthanized by cervical dislocation under anesthesia. Blood samples were collected in EDTA-2Na-containing 1.5 mL tubes and then centrifuged at 5000 rpm and 4 °C for 10 min to obtain plasma. Organ sampling was conducted as previously described [13]. In brief, the entire small intestine was excised, and the duodenum, extending from the pylorus to the ligament of Treitz, was isolated and flushed with ice-cold phosphate-buffered saline (PBS [2.7 mM KCl, 1.76 mM KH_2_PO_4_, 137 mM NaCl, 10 mM Na_2_HPO_4_]). The proximal one-third of the jejunoileum was considered the jejunum, whereas the remaining part was considered as the ileum. The central parts of the jejunum and ileum (approximately 1 cm) were histochemically stained. Thereafter, the colon was collected, and approximately 1 cm of the central part of the colon was used for morphological analysis. The remaining jejunum was used for immunoblotting. Samples were frozen in liquid nitrogen and stored at −80 °C until further analysis.

### 2.3. Histochemical Staining and Immunohistochemistry

Paraffin-embedded tissue sections were prepared and stained with hematoxylin and eosin as previously described [12]. Antigen retrieval was also performed as previously described [12]. Subsequently, samples were permeabilized using 0.3% H_2_O_2_ prepared in methanol and blocked with 5% goat serum (Sigma-Aldrich, St. Louis, MO, USA) in PBS for 1 h at 20–25 °C. The GLP-2 antibody (1:100, A5009, ABclonal Technology, Woburn, MA, USA) was diluted in 10% goat serum in PBS and incubated with samples overnight at 4 °C. Samples were washed thrice with PBS and incubated with a secondary antibody (1:150, #7074, horseradish peroxidase-conjugated anti-rabbit IgG, Cell Signaling Technology, Danvers, MA, USA) for 30 min at 25 °C. Finally, samples were washed thrice with PBS and stained using the DAB stain kit (Nacalai Tesque, Kyoto, Japan). All images were captured using a microscope (BZ-X710, Keyence, Osaka, Japan).

### 2.4. Measurements of Blood Lactate and Plasma GLP-2, Glucose, Triglyceride (TG), and Non-Esterified Fatty Acid (NEFA) Levels

Blood lactate levels were measured before and after exercise using Lactate Pro2 (LT-1730, ARKRAY, Inc., Kyoto, Japan). Total plasma GLP-2 levels were measured using a mouse GLP-2 EIA kit (Yanaihara Institute Inc., Shizuoka, Japan). Plasma glucose, TG, and NEFA levels were measured using Glucose C II-test Wako and lab assay kits (FUJIFILM Wako Pure Chemical Corporation, Osaka, Japan). All assays were performed according to the manufacturer’s instructions.

### 2.5. Immunoblotting

Total proteins were extracted from the jejunum using RIPA buffer (1 M Tris-HCl, 5 M NaCl, 0.5 M EDTA, 0.5% sodium dodecyl sulfate, 2.5% sodium deoxycholate, 5% NP-40) supplemented with proteinase inhibitor (cOmplete™ mini, Roche, Basel, Switzerland) and phosphatase inhibitor (PhosSTOP™, Roche, Basel, Switzerland)) tablets. Lysates were centrifuged at 15,000× *g* for 15 min at 4 °C. The concentration of total protein for each sample was measured using a BCA protein assay kit (Takara Bio, Shiga, Japan). Subsequently, 10 μg of total protein per lane was used for gradient gel electrophoresis. Proteins were transferred onto polyvinylidene fluoride (PVDF) membranes, which were then incubated with primary antibodies against SGLT1 (1:1000, A11976, ABclonal Technology, MA, Woburn, USA), GLUT2 (1:1000, 20436-1-AP, Proteintech Group Inc., Rosemont, IL, USA), GLUT5 (1:1000, 27571-1-AP, Proteintech Group Inc., Rosemont, IL, USA), GLP-2R (1:1000, A6602, ABclonal Technology, Woburn, MA, USA), and Sucrase-Isomaltase (1:1000, sc-393470, Santa Cruz Biotechnology, Dallas, TX, USA). Horseradish peroxidase-conjugated anti-rabbit IgG (1:5000, #7074, Cell Signaling Technology, MA, Danvers, USA) and anti-mouse IgG (1:5000, #7076, Cell Signaling Technology, MA, Danvers, USA) were used as secondary antibodies. Signals were detected using a chemiluminescence reagent (ECL Prime Western Blotting Detection Reagent, GE Healthcare, Chicago, IL, USA). Blots were scanned using a chemiluminescence imaging system (FUSION FX7.EDGE, Vilber Lourmat, Marne-la-Vallee, France).

### 2.6. Statistical Analysis

Data are presented as the mean ± standard deviation (SD). Data were subjected to unpaired *t*-tests in order to evaluate statistical significance. A value of *p* < 0.05 was considered to indicate significant difference. Statistical analyses were performed using GraphPad Prism 8.4.3 for Mac (GraphPad Software, San Diego, CA, USA).

## 3. Results

### 3.1. Acute Exercise Did Not Affect the Blood Lactate Levels

**Figure 1:**
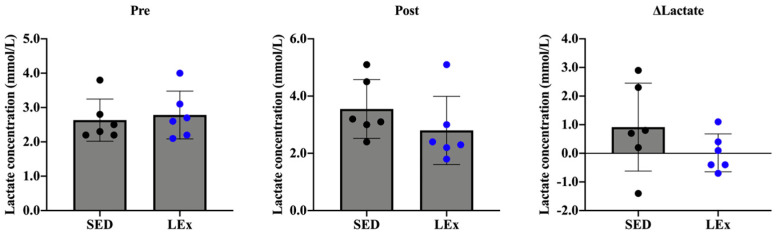
Effects of acute exercise on blood lactate concentrations. The concentration of blood lactate was measured before and after exercise. ΔLactate indicates the change in blood lactate concentrations before and after exercise. SED: sedentary group (*n* = 6). LEx: low-intensity exercise group (*n* = 6).

As low-intensity exercise was considered to be acute exercise, we measured the levels of blood lactate to validate exercise intensity. We did not observe any change in blood lactate concentrations (Figure 1).

### 3.2. Acute Exercise Increased Plasma GLP-2 Levels and Upregulated GLP-2R Expression in the Jejunum

**Figure 2:**
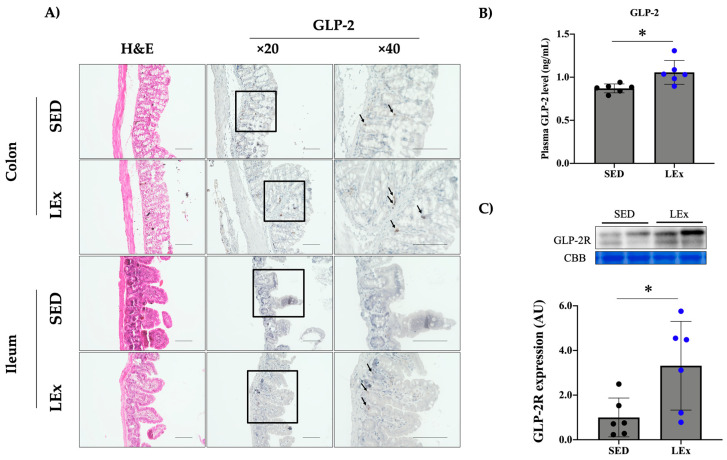
Effects of acute exercise on GLP-2 and GLP–2R. (**A**) Hematoxylin and eosin staining and GLP-2 immunohistochemistry. Scale bar, 100 μm. Black arrows indicate GLP-2. (**B**) Levels of plasma GLP-2. (**C**) Expression of GLP-2R in the jejunum. GLP-2: glucagon-like peptide-2, GLP-2R: glucagon-like peptide-2 receptor. CBB: Coomassie brilliant blue stain. To ensure equal protein loading across lanes, membranes were stained with CBB. The CBB bands represent the 35–48 kDa region where various internal controls are present. SED: sedentary group (*n* = 6). LEx: low-intensity exercise group (*n* = 6). * *p* < 0.05.

We measured GLP-2 levels in the ileum, colon, and plasma using immunohistochemistry and EIA. In addition, we measured the expression of GLP-2R in jejunal samples. We observed GLP-2 expression in the ileum and colon (Figure 2A). Further, plasma GLP-2 levels were higher in the LEx group (Figure 2B). The jejunal expression of GLP-2R was also upregulated in the LEx group (Figure 2C).

### 3.3. Acute Exercise Did Not Affect the Plasma Concentrations of Glucose, TG, and NEFA

**Figure 3:**
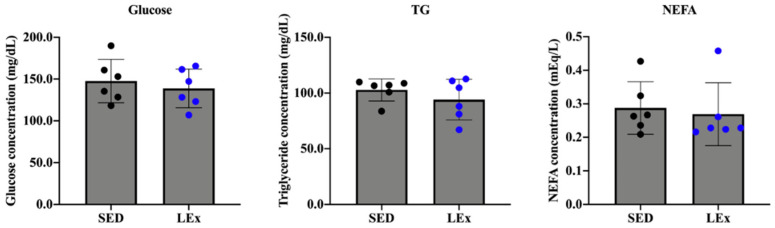
Effects of acute exercise on the concentrations of plasma glucose, TG, and NEFA. TG: triglycerides, NEFA: non-esterified fatty acids, SED: sedentary group (*n* = 6). LEx: low-intensity exercise group (*n* = 6).

To investigate the effect of acute exercise on major blood parameters, we measured the levels of glucose, TG, and NEFA in the plasma. We did not observe any change in these biochemical indexes (Figure 3).

### 3.4. Acute Exercise Upregulated the Expression of SI and GLUT2 Proteins in the Jejunum

**Figure 4:**
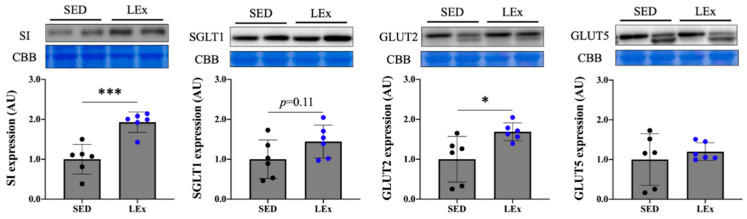
Effects of acute exercise on the expression of proteins related to carbohydrate absorption and digestion. *** *p* < 0.0005, * *p* < 0.05. SI: sucrase-isomaltase. SGLT1: Na+-dependent glucose transporter 1. GLUT2: glucose transporter 2. GLUT5: glucose transporter 5. CBB: Coomassie brilliant blue stain. To ensure equal protein loading across lanes, membranes were stained with CBB. The CBB bands represent the 35–48 kDa region where various internal controls are present. SED: sedentary group (*n* = 6). LEx: low-intensity exercise group (*n* = 6).

To investigate the effect of acute exercise on the digestion and absorption of carbohydrates, we assessed carbohydrate transporter and disaccharidase expression. We found that the expression of both SI and GLUT2 increased in the LEx group (Figure 4).

## 4. Discussion

This study explored the effects of acute exercise on the expression of GLP-2 and carbohydrate digestion/absorption-related molecules in the intestine. Exercise is known to enhance the formation of intestinal tight junctions [10], but its effect on the digestive and absorptive capability of the intestine is less well-known. To assess this, we subjected mice to a regimen of acute exercise and examined changes in the expression of disaccharidase and monosaccharide transporters as well as their positive regulator GLP-2 within the intestine.

We did not detect any changes in the pre- and post-exercise concentration of blood lactate (ΔLactate). Changes in blood lactate levels are known to be dependent on the intensity of exercise. Therefore, we confirmed that the exercise regimen employed in this study was of low intensity. High-intensity exercise is generally not feasible for patients (e.g., TPN or post-bowel removal patients). In contrast, patients may be able to perform low-intensity activities, such as walking. Therefore, the current results provide an incentive for the clinical introduction of low-intensity exercise for therapy.

We hypothesized that exercise would upregulate GLP-2. Accordingly, we observed an increase in plasma GLP-2 levels in the LEx group (Figure 2B). Only one study has investigated the changes in GLP-2 levels after exercise. Janssen Duijghuijsen et al. reported that the serum GLP-2 levels in well-trained healthy male cyclists increased after two exercise sessions [29]. However, their protocol included the intake of casein protein and a multisugar solution. As the secretion of GLP-2 is known to be increased by nutrient stimulation, i.e., through glucose and fat [30], the levels of GLP-2 could have been affected by the intake of casein protein and multisugar solution. In our study, mice were not fed after exercise. Therefore, to the best of our knowledge, this is the first study to report an increase in the levels of GLP-2 due to low-intensity exercise alone. However, the question remains as to how the release of GLP-2 was induced. We measured blood glucose, TG, and NEFA levels to assess whether low-intensity exercise affected carbohydrate or lipid metabolism. Changes in blood glucose, TG, and NEFA levels may occur during prolonged exercise due to increased energy demands. Such changes would affect intestinal digestion and nutrient absorption capability. If the fasting period is prolonged, the expression of intestinal epithelium-specific factors such as SI is markedly reduced [13]. However, following the low-intensity exercise regimen applied herein, no changes were observed in the abovementioned blood parameters (Figure 3). Therefore, the regulation of intestinal epithelium-specific molecules was independent of nutritional status and could be attributed to low-intensity exercise. GLP-2 is produced from proglucagon in L cells via the activity of prohormone convertase 1/3 (PC1/3) [30]. Few studies have explored the effects of exercise on PC1/3. Hiramoto et al. reported that moderate-intensity exercise (20 m/min, 1 h) upregulated PC1/3 in the pituitary gland [31]. Although intestine-specific expression changes were not assessed in this report, there is a possibility that PC1/3 expression or activity was enhanced within the intestine as well.

The nervous system may also be involved in the increase of GLP-2 secretion. It has been reported that the secretion of GLP-1, produced in the same manner as GLP-2, is upregulated via vagus nerve stimulation [32]. Therefore, stimulation of the vagus nerve may also drive increased GLP-2 secretion. Exercise is known to activate the sympathetic nervous system in an intensity-dependent manner. Brenner et al. reported that low––intensity exercise stimulates the parasympathetic nervous system [33]. Therefore, it is possible that the low-intensity exercise stimulated the vagus nerve, one of the representative parasympathetic nerves, in turn stimulating GLP-2 secretion.

The expression of the GLP-2R in the jejunum increased in parallel to GLP-2 levels. A number of carbohydrate digestion and absorption factors are known as downstream targets of GLP-2 signaling via GLP-2R. In particular, we found that the expression of SI and GLUT2 proteins increased significantly, while SGLT1 also showed an increasing trend, although not significant (Figure 4). GLP-2 has been previously reported to upregulate the expression of SI, which is known to be suppressed by TPN [18]. Au et al. observed that the jejunal expression of GLUT2 was promoted by GLP-2 in rats [16]. In addition, Cheeseman et al. found that administration of GLP-2 enhanced glucose uptake and SGLT1 expression in the rat jejunum [19]. Therefore, we assumed that the exercise-induced increase of GLUT2 and SGLT1 in this study might be mediated via GLP-2 signaling. Six weeks of swimming training were reported to upregulate SGLT1, deemed as an adaptative response of the intestine to long-term exercise [15]. The present study explored the effects of a 1 h treadmill run as a form of acute exercise. In previous studies, GLP-2 secretion was increased during and after exercise, with a subsequent increase in GLUT2 protein expression, and a trend toward increased SGLT1 expression is also reported [16,19]. In addition, as an increase in transporter protein expression was observed an hour after GLP-2 administration, we speculate that the SI expression may also have been increased by GLP-2. As treadmill running and swimming are distinct forms of exercise, they cannot be accurately compared. Nevertheless, GLP-2 might be a key factor in intestinal adaptation to exercise.

The current study had certain limitations. There are two types of GLP-2 in blood, namely active (GLP-2^1–33^) and inactive (GLP-2^3–33^). Herein, we measured was the total amount of both together. Thus, we did not assess exercise-induced changes in the active form of GLP-2, which should be addressed in future research. In addition, experiments utilizing long-term training by treadmill running would help elucidate the relationship between GLP-2 and exercise. Another limitation is that we could not directly measure increases in digestion and absorption capacity. In future studies, we will obtain greater insight by measuring the amount of glucose uptake through the use of live imaging or radioisotopes. Finally, we did not confirm that the observed increases in SI and GLUT2 expression were due to GLP-2 alone. To this end, we need to employ GLP-2R KO mice and assess whether the observed effect of exercise is compromised. Overcoming these limitations in future studies will allow us to further substantiate the use of low-intensity exercise therapy for increasing levels of GLP-2, a key molecule associated with the digestion and absorption of carbohydrates within the small intestine.

To date, studies on the effects of exercise on the small intestine have mainly focused on the regulation of tight junctions. In the current study, we report the effects of exercise on carbohydrate digestion and absorption within the small intestine, as carbohydrates constitute one of the major nutrient sources for humans. As the intestine is atrophied in TPN and short bowel syndrome, the restoration of intestinal function is of major importance in these patients. Taken together, the current findings suggest that low-intensity exercise might contribute to the recovery of intestinal function by upregulating GLP-2 levels. In the future, the effects of exercise on intestinal function should be further clarified using TPN and short bowel syndrome animal models.

## 5. Conclusions

Herein, we examined the effects of acute low-intensity exercise on GLP-2 levels as well as the intestinal expression of molecules involved in carbohydrate digestion and absorption. Exercise enhanced GLP-2 secretion and consequently the expression of SI and GLUT2. Hence, exercise might improve the digestion and absorption of carbohydrates in the small intestine by increasing GLP-2 levels.

## Data Availability

Data can be made available on request.
